# Bibliography

**Published:** 1900-11

**Authors:** 


### Bibliography.

Facts, Fads, and Fancies about Teeth. Compiled and Edited by Henry Lovejoy Ambler, M.S., D.D.S., M.D., Dean of Dental Department and Professor of Operative Dentistry and Dental Hygiene, Western Reserve University, etc. Illustrations by W. L. Evans. The Helman-Taylor Company, Cleveland, 1900.

This book of three hundred and ten pages has been compiled by the editor with the object of preserving "the witty and humorous sayings gathered from all sources while at home and abroad, during many years of professional life." For those who love jokes and prefer to always live in an atmosphere of jollity, this book will be found a mine of good things, but to those who regard professional work as worthy the most dignified treatment, this collection will not appeal. While it is true that it is not wise to dwell continually upon the serious side of life, it is equally true that he who would elevate his profession must avoid giving countenance to anything that would lower the tone of those who practise it.

It has been the work of many decades to raise the dental profession from the ill-disguised contempt involved in the expression "tooth-puller." To give, therefore, the poor jokes of the "space writer" a permanent setting is simply lowering the dental profession to the level of this original standard set up by the laity of that period. Why endeavor to perpetuate these things and add to them pictures well fitted for *Puck?* The writer does not question the motive of the editor. Judging from his stand-point, his work is worthy of commendation, but from the professional it is extremely distasteful. If we are to rise worthy of the honors the doctorate confers, these squibs of a really contemptuous age must be consigned to oblivion.

Having written this much by way of criticism, it is a pleasure to turn to the latter part of the book, under the title "History of Dentistry and Miscellaneous Items." Here are many interesting paragraphs and quotations collected that might profitably be extended.

The attention of the editor is called to a seeming error on page 227, where he states that "In 1844 S. S. White began the production of porcelain teeth in Philadelphia." This is true so far as the foundation of that great house is concerned, but it leaves the impression that the manufacture of these teeth commercially began then. Samuel W. Stockton, some years prior to this date,—1836,—was a large manufacturer of porcelain teeth. He was an uncle of Samuel Stockton White, and the latter was originally engaged in the manufacture with the former.

The illustrations of the book, judged by the humorous standard, are extremely good and exhibit more real wit than any portion of the text.

Notes on the Treatment of Irregularities in Position of the Teeth. By J. F. Colyer, L.R.C.P., M.R.C.S., L.D.S., Dental Surgeon to the Dental Hospital of London, Dental Surgeon to Charing Cross Hospital, and Lecturer on Dental Surgery in the Medical School. Published by The Dental Manufacturing Co., Limited. London, 1900.

There is no better evidence of the progress made in dentistry than the fact that in the last decade the number of books upon special subjects have multiplied to an extent that it is now possible to possess a respectable library covering all the branches into which dentistry has been subdivided. In no one of these has there been more rapid development than in orthodontia.

The subject-matter of this work of the author "formed the basis of a series of lectures given to dental students at Charing Cross Hospital during the early part of last year." The book is profusely illustrated with skiagrams and engravings, leaving little to be desired in this respect.

The author, in discussing the age of the patient, says, as we all know, that "the difficulty of moving and retaining the teeth in a new position, increases with the age. As a rule, it is not advisable to attempt correction of irregular teeth by mechanical means in adults." While the difficulty of moving teeth increases with age, it seems unreasonable to confine regulation to children.

The methods described on pages 26 and 27, in which rubber and wood are recommended, carries one back to a period when these were considered indispensable in the movement of a certain class of irregularities; but why illustrate the manner of use now when better methods in the application of force have taken their place?

There is probably no branch of dental work where a wider latitude of opinion and practice may be tolerated than in the regulating of teeth, and as this interesting and carefully prepared book is read, the temptation is constantly presented of stopping to criticise opinions and practice not consistent with the writer's judgment.

It is impossible, however, to agree with the author's views upon the extraction of teeth to correct irregularity. While this is recognized as a valuable aid in overcoming a difficulty, it should be made use of sparingly, and then only with well-considered judgment. The following quotations will illustrate the author's idea.

"The first permanent molars are filled or treated in the manner best calculated to retain them until the second permanent molars have erupted. The crowding of the upper and lower incisors is then relieved by the removal of the four deciduous canines. If the teeth erupt in the normal way, the first and second premolars will come into good position. . . . We shall have to deal with a fairly simple irregularity,—namely, the canines high in the arch. To make room for the canines the first permanent molars should be removed directly the second permanent molars are fairly through the gums." This seems to the reviewer as an effort to remove one irregularity by substituting another. The malposition of the second molar is practically assured by this practice, together with the entire destruction of normal articulation. In regard to treatment of "crowding resulting in the exclusion of laterals from the arch," the author gives this advice: "The advisability of sacrificing a lateral incisor in the treatment of crowding is constantly disputed. . . . The principal argument urged against the removal of this tooth is that the canine erupts next the central. . . . Is it better to have a canine in correct alignment next to the central and the premolar in apposition to the canine or lateral in a mal-direction, with, possibly, its cutting edge tilted forward and the canine sloping towards the median line, and in all probability short?" The illustration given represents the two laterals immediately posterior to the canines and centrals, with considerable space between centrals and canines. If extraction must be resorted to in such presentations, the removal of the first premolar would be proper in order to save the lateral. There is no disfigurement more pronounced, especially in a woman, than canines aligned with centrals.

Space does not permit further notice of this book. It seems to the reviewer to lack systematic treatment, and shows a poverty of mechanical appliances somewhat surprising. It certainly in this respect compares unfavorably with works by American authors. The illustrations are one of the best features of the book and clearly indicate the points explained in the text.

While the reviewer is forced to differ frequently with the teaching of this author, he recognizes it as a valuable addition to the literature of the subject. It offers methods of comparison between the practice regarded as satisfactory in the dentistry of England and that regarded as producing the best results in this country.

The care with which this book has been prepared, both in illustrations, press-work, and type, merits positive commendation. It is a satisfaction as well as a pleasure to read and review works in which all details are carefully considered.

